# The Dimorphos ejecta plume properties revealed by LICIACube

**DOI:** 10.1038/s41586-023-06998-2

**Published:** 2024-02-28

**Authors:** E. Dotto, J. D. P. Deshapriya, I. Gai, P. H. Hasselmann, E. Mazzotta Epifani, G. Poggiali, A. Rossi, G. Zanotti, A. Zinzi, I. Bertini, J. R. Brucato, M. Dall’Ora, V. Della Corte, S. L. Ivanovski, A. Lucchetti, M. Pajola, M. Amoroso, O. Barnouin, A. Campo Bagatin, A. Capannolo, S. Caporali, M. Ceresoli, N. L. Chabot, A. F. Cheng, G. Cremonese, E. G. Fahnestock, T. L. Farnham, F. Ferrari, L. Gomez Casajus, E. Gramigna, M. Hirabayashi, S. Ieva, G. Impresario, M. Jutzi, R. Lasagni Manghi, M. Lavagna, J.-Y. Li, M. Lombardo, D. Modenini, P. Palumbo, D. Perna, S. Pirrotta, S. D. Raducan, D. C. Richardson, A. S. Rivkin, A. M. Stickle, J. M. Sunshine, P. Tortora, F. Tusberti, M. Zannoni

**Affiliations:** 1 Osservatorio Astronomico di Roma, INAF, Rome, Italy; 2 Dipartimento di Ingegneria Industriale, Alma Mater Studiorum, Università di Bologna, Forlì, Italy; 3 Osservatorio Astrofisico di Arcetri, INAF, Florence, Italy; 4 Observatoire de Paris, LESIA, Paris, France; 5 Istituto di Fisica Applicata ‘Nello Carrara’, CNR, Florence, Italy; 6 Politecnico di Milano, Milan, Italy; 7 Space Science Data Center, ASI, Rome, Italy; 8 Agenzia Spaziale Italiana, Rome, Italy; 9 Università degli Studi di Napoli ‘Parthenope’, Naples, Italy; 10 Osservatorio Astronomico di Capodimonte, INAF, Naples, Italy; 11 Osservatorio Astronomico di Trieste, INAF, Trieste, Italy; 12 Osservatorio Astronomico di Padova, INAF, Padova, Italy; 13 Johns Hopkins University Applied Physics Laboratory, Laurel, MD USA; 14 IUFACyT, Universidad de Alicante, Alicante, Spain; 15 Jet Propulsion Laboratory, California Institute of Technology, Pasadena, CA USA; 16 Department of Astronomy, University of Maryland, College Park, MD USA; 17 Centro Interdipartimentale di Ricerca Industriale Aerospaziale, Alma Mater Studiorum, Università di Bologna, Forlì, Italy; 18 Auburn University, Auburn, AL USA; 19 Space Research and Planetary Sciences, Physikalisches Institut, University of Bern, Bern, Switzerland; 20 Planetary Science Institute, Tucson, AZ USA; 21 Istituto di Astrofisica e Planetologia Spaziali, INAF, Rome, Italy

**Keywords:** Asteroids, comets and Kuiper belt, Astronomical instrumentation

## Abstract

The Double Asteroid Redirection Test (DART) had an impact with Dimorphos (a satellite of the asteroid Didymos) on 26 September 2022^1^. Ground-based observations showed that the Didymos system brightened by a factor of 8.3 after the impact because of ejecta, returning to the pre-impact brightness 23.7 days afterwards^2^. Hubble Space Telescope observations made from 15 minutes after impact to 18.5 days after, with a spatial resolution of 2.1 kilometres per pixel, showed a complex evolution of the ejecta^3^, consistent with other asteroid impact events. The momentum enhancement factor, determined using the measured binary period change^4^, ranges between 2.2 and 4.9, depending on the assumptions about the mass and density of Dimorphos^5^. Here we report observations from the LUKE and LEIA instruments on the LICIACube cube satellite, which was deployed 15 days in advance of the impact of DART. Data were taken from 71 seconds before the impact until 320 seconds afterwards. The ejecta plume was a cone with an aperture angle of 140 ± 4 degrees. The inner region of the plume was blue, becoming redder with increasing distance from Dimorphos. The ejecta plume exhibited a complex and inhomogeneous structure, characterized by filaments, dust grains and single or clustered boulders. The ejecta velocities ranged from a few tens of metres per second to about 500 metres per second.

## Abstract (web summary)

Dimorphos ejecta plume properties were revealed by the observations from the LICIACube cube satellite, which was deployed 15  days in advance of the impact of DART.

## Main

The Italian Space Agency (ASI) Light Italian Cubesat for Imaging of Asteroids (LICIACube)^6^ is a 6U CubeSat carried by the NASA Double Asteroid Redirection Test (DART) spacecraft and deployed on 11 September 2022, 15 days in advance of the impact of DART with asteroid Dimorphos^7^, to acquire images of the event and its effects.

**Fig. 1:**
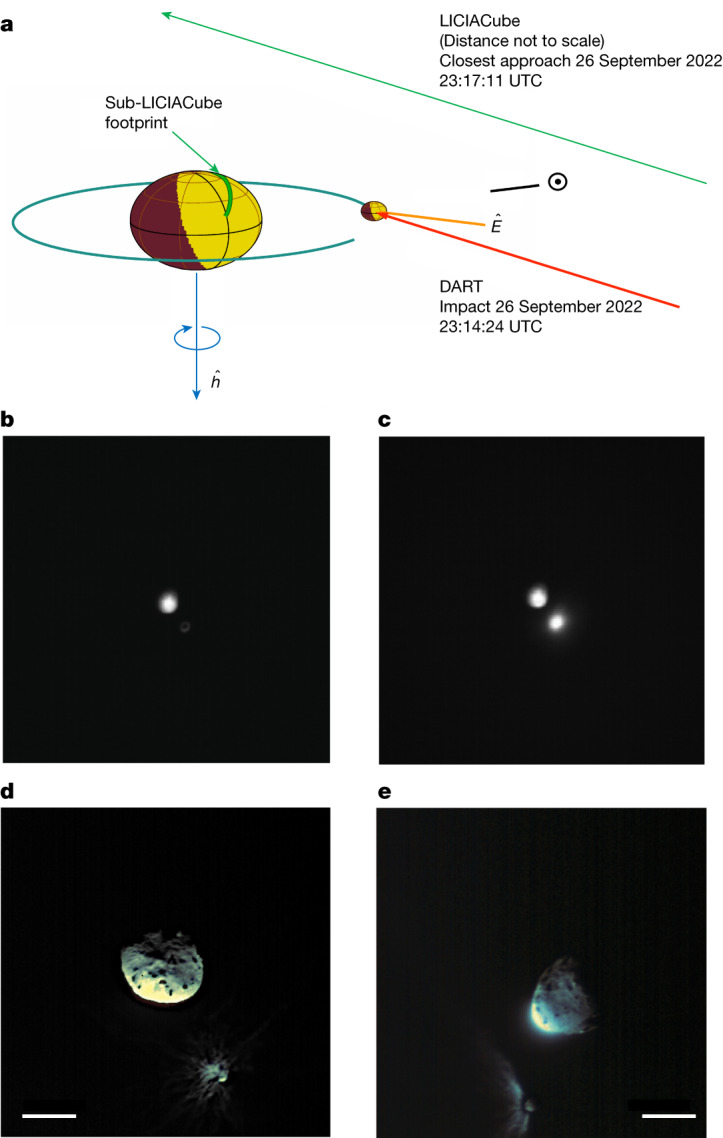
The viewing geometry of LICIACube. **a**–**e**, Schematic of the DART impact and LICIACube viewing geometries (**a**) and cropped images of Didymos and Dimorphos as seen by LEIA (**b**,**c**) and LUKE (**d**,**e**). **a**, The trajectory and the footprint on Didymos are shown in green; *ĥ* is the positive pole direction of Didymos; the red arrow is the incident direction of the DART; *Ê* is the cone axis pointing; the relative direction of the Sun is also shown in yellow. **b**, The binary system imaged at a distance of approximately 1,000 km, 5 s before the impact: Didymos is visible in the centre of the LEIA FOV and Dimorphos appears as a ring (because of the de-focusing of the instrument, discovered on flight) at the lower right side of Didymos. **c**, The same scene viewed 1 s after the impact: the expanding ejecta plume causes an increase of a factor 5, in terms of DN over the same area in the lower right side of Didymos. **d**, RGB image of the targets acquired at a distance of 76 km, 8.5 s before CA (159 s after impact). **e**, RGB image at a distance of 71 km, 6.5 s after CA (174 s after impact). Scale bars, 500 m (**d**,**e**).

During its post-impact fly-by (Fig. 1a), the probe acquired and returned 426 scientific images, obtaining a unique view of the event with phase angles ranging from 43° to 118°. Images were acquired with two instruments—the LICIACube Explorer Imaging for Asteroid (LEIA) and the LICIACube Unit Key Explorer (LUKE)^6^. The science phase began 71 s before the nominal impact time, when the small probe was 1,466 km from Dimorphos. In LEIA images, the DART impact caused an increase in intensity by approximately a factor of 5, in terms of digital counts (DN) integrated over a fixed area pre- and post-event (Fig. 1b,c). The scientific phase of LUKE started 29 s after the impact, acquiring triplets of images with different exposure times. Both instruments followed the target and the evolution of the system up to 320 s after the impact (23:14:24.183 ± 0.004 UTC)^1^. The closest approach (CA) occurred about 167 s after the impact, at a distance of about 58 ± 2 km from Dimorphos (Fig. 1a). In the spacecraft viewing geometry, ejecta produced by the impact were clearly seen in both pre-CA and post-CA images (Fig. 1d,e). In the post-CA geometry (Fig. 1e), there is a dark arc between the bright plume and Dimorphos because of a shadow cast by the optically thick plume.

**Extended Data Fig. 1:**
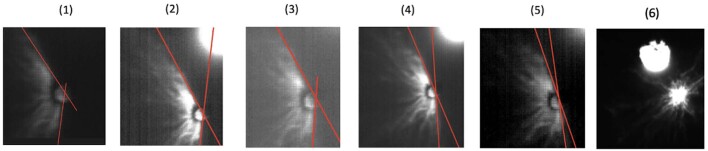
The ejecta curtain as seen by LICIACube. The cropped parts of images that were used for the analysis. The red lines in panels 1–5 indicate the slant axes of the ejecta cone that were used to measure the projected aperture angle 2δ. Panel 6 shows the ejecta cone in a different geometry than those in panels 1–5, which was used as a discriminator when accepting or rejecting candidate solutions for the ejecta cone. Image IDs correspond to those given in Extended Data Table 1.

**Extended Data Fig. 2:**
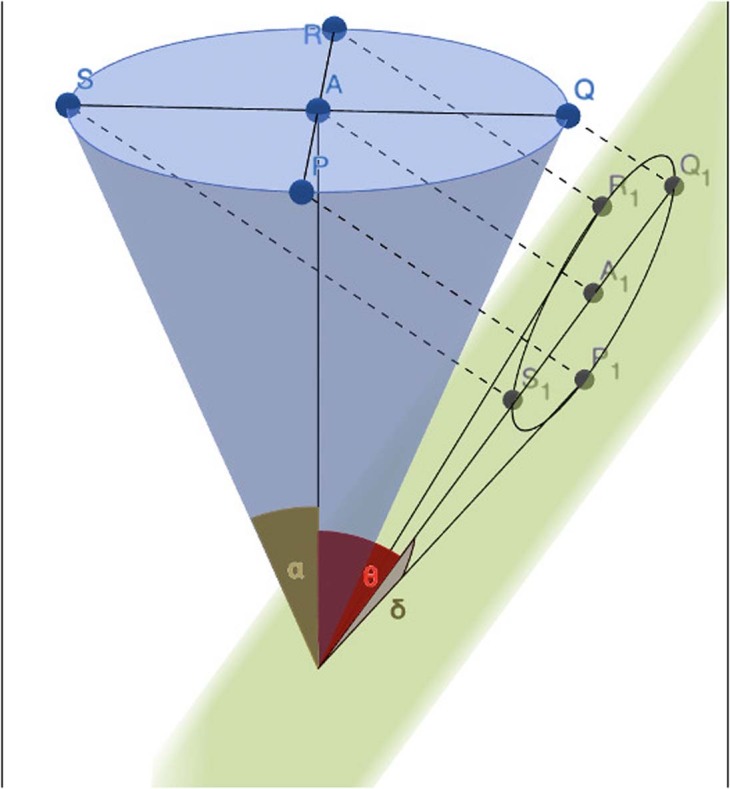
The ejecta cone geometry. A perfectly axisymmetric cone and its projection onto a plane which gives the relation between the original cone aperture angle and the aperture angle of the projected cone given in Eq. (1). In the plot, the original cone is sliced perpendicularly to its axis. On the slice, the centre along the cone axis is defined as A, and the intersections of the two orthogonal axes with the cone shell are also denoted as P, Q, R, and S. Their projected points on the plane are given using subscript 1.

**Extended Data Fig. 3:**
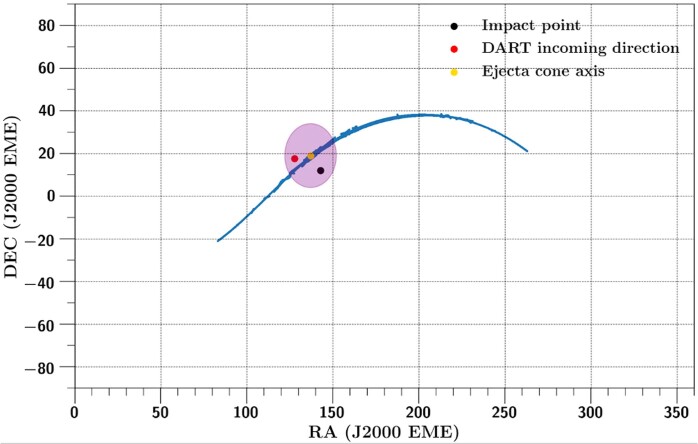
Ejecta cone orientation. The blue curve, obtained by matching the position angle of the ejecta cone in image ID1, corresponds to the solutions of the ejecta cone axis direction that are mathematically possible. The yellow dot indicates our solution of RA, DEC: 137°, + 19° constrained by comparing the simulated ejecta cones with the LUKE images in Extended Data Fig. 1 and its uncertainty region is illustrated by the transparent purple circle. DART incoming direction (just before the impact) of RA, DEC: 127°, + 18° and the impact point at RA, DEC: 143°, + 12° are given as references.

We characterize the axis and the aperture angle of the observed ejecta cone from the images, using the assumption that the ejecta cone is axisymmetric. Six images (Extended Data Table 1) were used in the analysis. The ejecta cone is seen in a projected side-on in the five post-CA images and in a projected head-on profile in the one pre-CA image (Extended Data Fig. 1). The aperture angle and the axis of the cone are retrieved on the basis of geometric considerations (see Methods and Extended Data Fig. 2 for details). The solution is a cone with an aperture angle of 140 ± 4° with its axis pointing to (right ascension (RA), declination (DEC) in J2000 frame) $=\,{{137}_{-9}^{+8}}^\circ ,\,+{{19}_{-12}^{+10}}^\circ$), consistent with refs. ^2,3^ (Extended Data Fig. 3). This aperture angle is slightly wider than the one computed using Hubble Space Telescope (HST) images^3^, possibly because of asymmetric features seen in the different viewing geometries. If the cone is axisymmetric, this analysis suggests that the surface of Didymos could be marginally intercepted by material directly ejected from Dimorphos, whereas dynamical evolution of the slow ejecta could bring materials to Didymos over time (see, for example, ref. ^8^).

**Extended Data Fig. 4:**
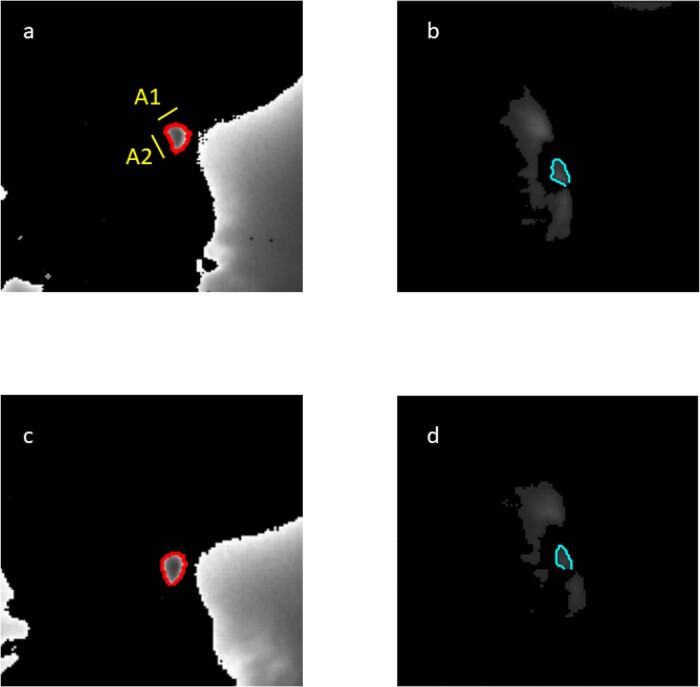
The shape of Dimorphos. The two pairs of images used to identify the illuminated and non-illuminated hemispheres of Dimorphos and compute the size. Images at frames (a) liciacube_luke_l2_1664234241_00417_01 and (c) liciacube_luke_l2_1664234244_00417_01 have an exposure time of 0.035 s; images at frame (b) liciacube_luke_l2_1664234241_00007_01 and d (liciacube_luke_l2_1664234244_00007_01) of 0.0007 s. The red shape in (a) and (c) identifies the non illuminated hemisphere (with areas of 5330 and 5320 m^2^, respectively and accuracy of 60 m^2^), whereas the cyan shape in b and d identifies the illuminated one (with areas of 3100 and 2220 m^2^, respectively and accuracy of 200 m^2^). A “dark arc” is evident between this shape and the plume. A1 and A2 in image (a) indicate the two axes identified and described in the text.

LICIACube imaged Dimorphos with a different viewing geometry than DART and further constrained the size and shape of Dimorphos itself. Applying computer vision algorithms^9^ to images with different exposure times, the non-illuminated cross-sectional area of the non-impacted hemisphere (Methods and Extended Data Fig. 4) is around 5,300 m^2^ (with an uncertainty of about 2 pixels square, that is, 200 m^2^), in agreement with what is expected by using axis dimensions retrieved from DART images^1^.

**Fig. 2:**
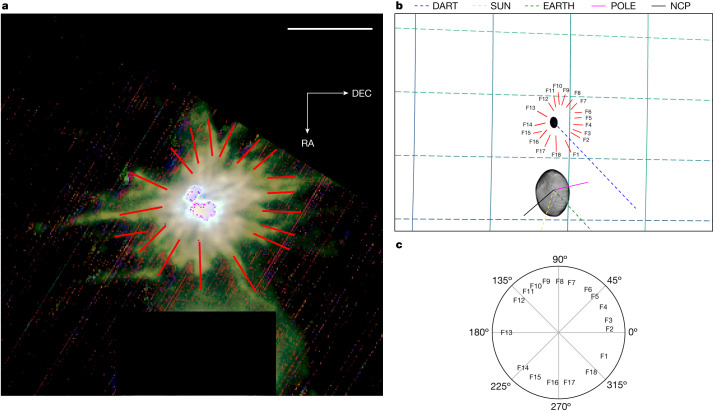
Identification and orientation of the filamentary streams. The directions of the streams are computed as they extend from the surface of Dimorphos. **a**, Filaments are superimposed onto a LUKE RGB composition of the image triplet obtained at 2022-09-26T23:16:58.916, *T* + 154 s. The spatial resolution of the image is 7.5 m per pixel at 97 km from Dimorphos. The filaments are counted at projected distances higher than 230 m from the photocentre of the ejecta. The frame has been rotated and recentred with respect to Dimorphos. Dimorphos is saturated and Didymos has been masked. Scale bar, 0.5 km. **b**, Supporting synthetic frame with the binary system and the filaments superimposed onto the RA/DEC sky plane (green grid). DART, incoming DART spacecraft vector; SUN, solar vector; EARTH, vector of Earth; POLE, Didymos system rotation pole vector; NCP, North equatorial celestial pole vector. Shape models of Didymos and Dimorphos from ref. ^18^ and ref. ^1^, respectively. **c**, Angular orientation of the filaments with respect to DART incoming velocity vector in the RA/DEC sky plane.

**Fig. 3:**
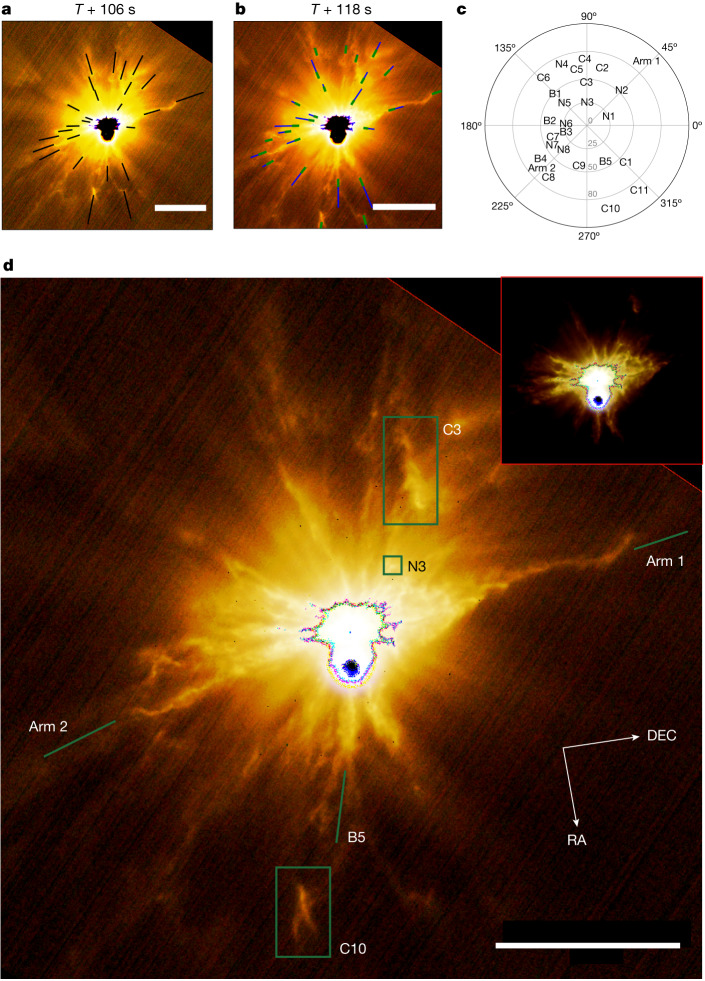
Measured ejection velocities of some morphological features in the ejecta plume. Many complex structures can be noted (see the full list in Extended Data Table 2): the two arm-like streams arising from F5/F6 and F14/F15 (identified in Fig. 2); resolved detached structures, named clumps (C); bright punctiform-like unresolved features, named nodules (N); filament breaking, merging, discontinuities and undulations (B). **a**, Frame obtained 106 s after the impact superimposed with the projected displacement measured between two frames (black lines). **b**, Frame obtained 118 s after the impact superimposed with the two solutions for FOV-corrected magnitude velocities (blue and green vectors, respectively, see Methods). **c**, Orientation and projected velocities in the RA/DEC sky plane and angle with respect to the DART incoming velocity vector. **d**, RGB composition of LUKE triplet images obtained 118 s after the impact. The spatial resolution of the image is 23 m per pixel at 304 km from Dimorphos. All the frames have been rotated and recentred with respect to Dimorphos. Didymos and Dimorphos are saturated. Scale bars, 5 km (**a**,**b**,**d**).

Filamentary streams, as well as many complex patterns, are observed to expand for several kilometres from Dimorphos (Figs. 2 and 3), suggesting collimated radial outflows. The emergence of these streams near the surface becomes evident at 154 s after the impact (CA-13s) in which the inner about 250 m within the ejecta reveals 18 main filaments in the image (Fig. 2). By examining the triplet of images taken 36 s earlier (*T* + 118 s, CA-49s) it is possible to track the expansion of filaments from 0.5 km to 8.8 km, discerning their morphological evolution (Fig. 3). Two diametrically opposed thin streams ((F5, F6) and (F14, F15)) are evident, evolving into long arm-like structures with curving ends extending for 6–8 km from Dimorphos. Both structures are persistent and are present since the first frame. Measurements between the two RED channels (*T* + 106 s and *T* + 118 s) indicate projected radial velocities of 67 m  s^−1^ and 47 m s^−1^, respectively.

Other fast persistent structures were witnessed, such as a clump of material (C10) observed for 96 s and ejected at a projected radial velocity of about 75 m s^−1^ aligned to F17 (Fig. 3). Some visual detachments of optically thick material are evident as, for example, a bright clump (C3) with a projected radial velocity of 29 m s^−1^ coming off the F10 stream between 34 s and 46 s after the impact. Many undulating patterns seem to be azimuthally connected with filamentary streams, as F1, F14, F17 and with both arm-like structures. These filaments experienced discontinuities and bifurcations at different distances. Bright nodules along many of the streams, especially between F4 and F10 as well as between F13 and F15, may be evidence of larger ejected components (for example, single blocks, boulder clusters). Diffused clumps are also noticeable as resolved detached structures, possibly related to aggregates of particles or to large grains in the process of fragmentation.

**Extended Data Fig. 5:**
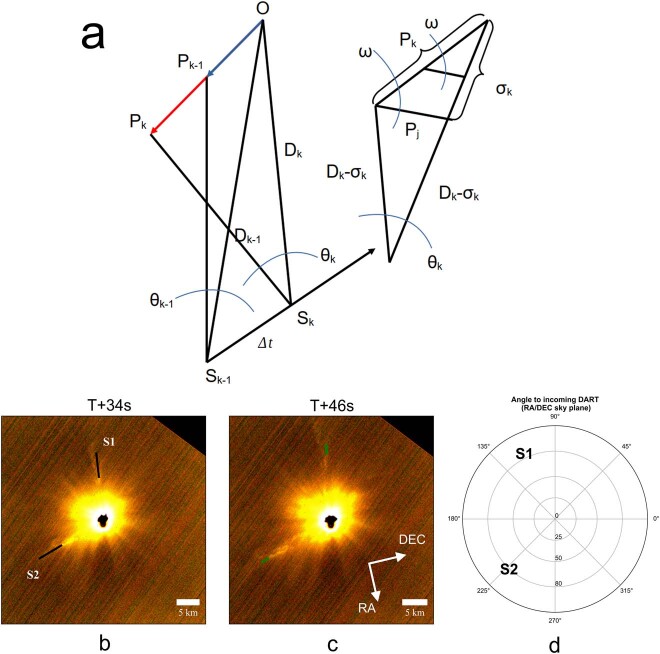
The early resolved ejected structures. (a) Scheme of the Pythagorean solution for the projection-correction of ejecta velocity assuming that the projection angle ω is virtually the same for the two frames taken at short time difference and large observer distance to the ejecta structure, which is displacing from its origin at the time of the impact. The scales are exaggerated for better visualisation. The labels are: O – origin, k – frame index; S – spacecraft position; P – Position of the ejecta structure; P_j_ – structure extension projected to frame; D – distance from spacecraft to origin; θ – angular distance to of structure to origin measured from spacecraft; σ – distance between origin and projection P_j_ on the frame plane. (b,c,d) Velocities from early resolved ejected structures from the pair of LUKE frames obtained 34 and 46 s after the impact. (b): 34-seconds-after frame is superimposed with the measured projected displacements in black. (c): 46-seconds-after frame is superimposed with the FOV-corrected velocity magnitudes in green. (d): The orientation and projected velocity magnitudes in the RA/DEC sky plane with respect to the DART incoming velocity vector. The frame has been rotated and recentered with respect to Dimorphos. Didymos and Dimorphos are saturated.

The velocities of the resolved morphological features pre-CA (Fig. 3, Methods and Extended Data Table 2) are of the same order of magnitude as those of the inner boundary ejecta produced by Deep Impact on comet 9P/Tempel 1 (around 80 m s^−1^) (ref. ^10^). The most distant plume structures in the earliest images are the best indicators of the first optically thick ejected materials and the highest ejection velocities (Extended Data Fig. 5). Two faint non-saturated structures were resolved at 34 s after the impact and tracked afterwards: (S1) one radial-linear filament at 15.4 km from Dimorphos and 1.5 km in length; and (S2) a co-moving spiral-like cluster of optically thick components at 11.7 km and 3.2 km in length, respectively. S1 is tracked through two frames after impact, resulting in a radial velocity of 420–490 m s^−1^, whereas S2 is tracked through three frames, giving a lower velocity of 290–400 m s^−1^, after field of view (FOV)-projection corrections (Methods and Extended Data Table 3). These measurements are one order of magnitude larger than the highest velocities (projected onto the telescope view plane) reported by HST observations about 2 h after the DART impact^3^, while they are consistent with the highest velocities of the ejecta observed by HST immediately after the Deep Impact experiment (approximately 300 m s^−1^) (ref. ^10^).

**Fig. 4:**
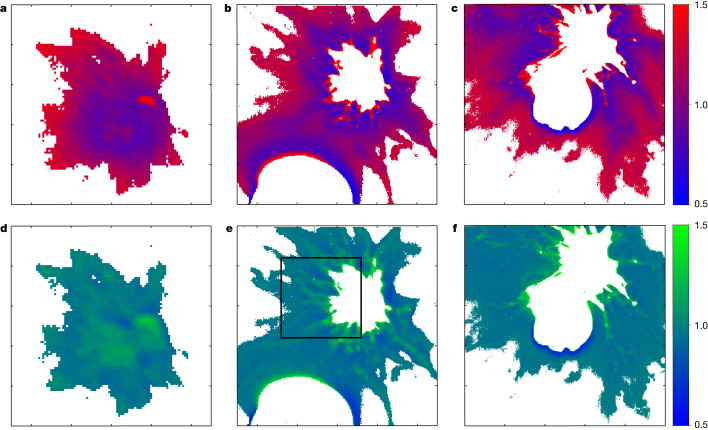
Flux ratios in LUKE colour filter data for image triplet. **a**–**f**, Images with different exposure times (nominally 0.5 ms for **a** and **d**, 4 ms for **b** and **e**, and 20 ms for **c** and **f**). The colour bars are the red:blue (**a**–**c**) and the green:blue (**d**–**f**) ratios. The black rectangle in **e** highlights specific details of the plume structure with visible streams characterized by a green colour over the average blue colour of the inner part of the plume (see the text for details).

**Extended Data Fig. 6:**
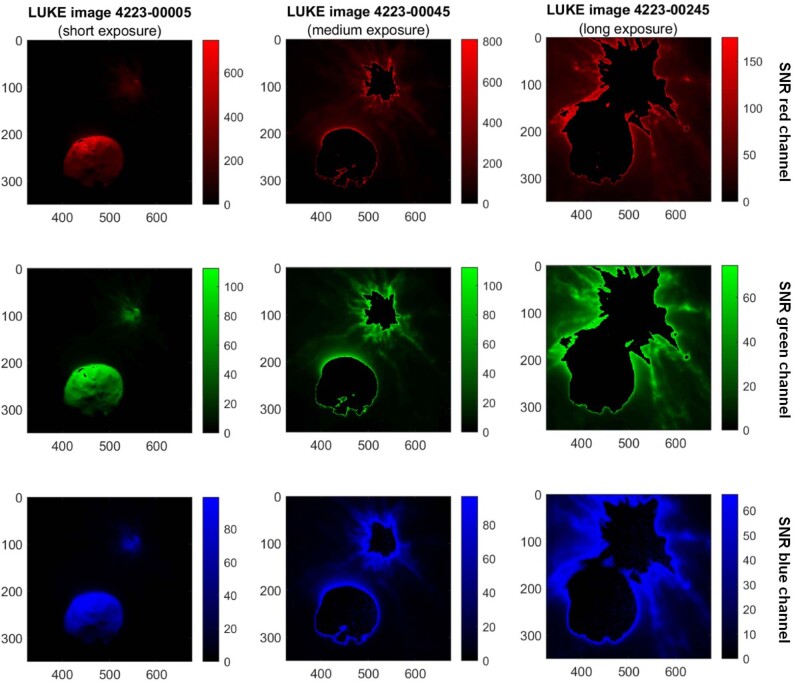
Signal to Noise values for RGB channels in LUKE images. The showed S/N have been evaluated from background of images acquired at time 2022-09-26T23:17:03.000Z (0.5 ms exposure time, SCLK 1664234223), 2022-09-26T23:17:03.004Z (4 ms exposure time), and 2022-09-26T23:17:03.024Z (20 ms exposure time).

**Extended Data Fig. 7:**
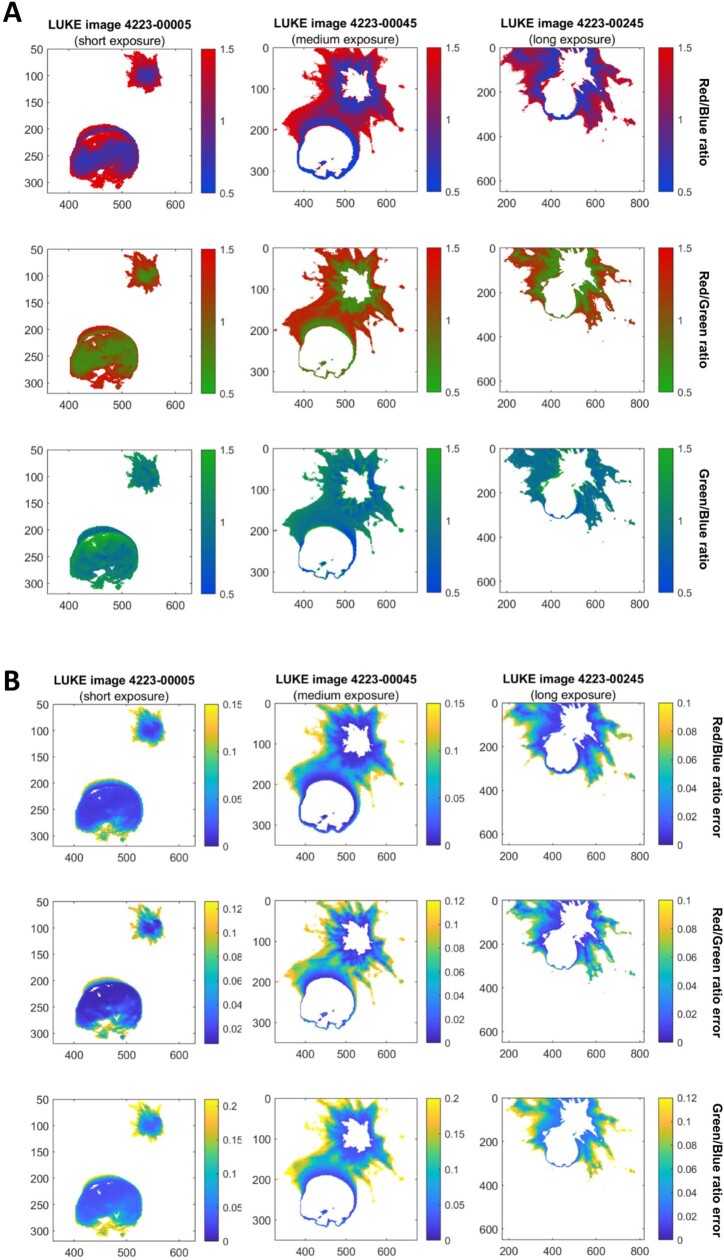
Didymos-Dimorphos maps of flux ratio in LUKE colour filter data. Flux ratio (panel A, top) and fluxes ratio error (panel B, bottom) for LUKE images at different exposure times (see RGB analysis method text for reference). From the top row to the bottom: red over blue ratio, red over green ratio and green over blue ratio.

The flux ratios of the red and blue channels for a selected triplet acquired pre-CA (refer to Methods for the images times and masking process) with integration times of 0.5 ms, 4 ms and 20 ms are shown in Fig. 4a–f. For each image, using the three colour planes captured by the RGB filters, red:blue and green:blue flux ratios are evaluated (refer to Methods and Extended Data Figs. 6 and 7 for signal-to-noise values for RGB channels and maps of flux ratio with relative uncertainties). Long-exposure images (4 ms and 20 ms) exhibit the saturation of the centre of the plume, while allowing the study of the outermost parts. The inner region is characterized by a blue colour (Fig. 4, left); nevertheless, it is clear that the plume progressively becomes spectrally red with increasing distance from Dimorphos. This is also observed in the longer exposure images (Fig. 4b,c). The average flux ratio of the red and blue channels from the inner part of the plume in the medium exposure image is 0.57, whereas the outer part is characterized by an average ratio of 0.96.

There are different possible explanations for the observed colour variations. The blue colour in the inner ejecta plume could be related to abundant sub-µm dust grains, as seen in the Deep Impact experiment (see ref. ^11^ and references therein). Alternatively, the colour difference could be because of redder surface material altered by space weathering^12^ being ejected first in the DART impact, with less-altered and bluer subsurface material ejected later. It is not clear, however, whether the amount of ejected surface material is enough to explain this colour difference. Reddening observed in the outer part of the plume of comet 73P/Schwassmann-Wachmann 3 (ref. ^13^) was ascribed to silicate fragmentation possibly because of electrostatic disruption, thermal stress, grain acceleration, vaporization of an organic component or centrifugal forces. These phenomena are invoked when the physics of cometary comae and striae is retrieved from observations (see refs. ^14–17^ and references therein).

Observing the green:blue ratio (Fig. 4d–f), the ejecta plume does not show a strong difference between the inner and the outer parts. The filamentary streams in the inner ejecta seem to have a green colour that stands out over the bluer background of the inner part of the plume, indicating again a difference in the physical characteristics of the material composing them (Fig. 4e, area highlighted by the rectangle).

The structure of the analysed cone is irregular, if compared with previous studies and simulations that used the Moon and/or DI/Tempel 1 comet tests. The colours of the Dimorphos ejecta suggest that even if the plume might have a homogeneous composition, filaments can have different colours possibly because of varying physical characteristics and/or alteration in the ejected materials.

## Methods

### LUKE image calibration process

During the ground activities of the integration and test phases of the LICIACube, several sessions of calibration measurements were carried out to fully characterize the performances of the instruments. Measurements were taken both with and without external calibrated light sources.

The acquisition of images in dark conditions enabled the characterization of the electrical parameters of the detector. Dark current, fixed pattern noise and readout noise of the detector and their dependence on the temperature for each pixel were characterized and measured.

The calibration curves for radiance and digital counts (DN) of the instruments were obtained by measurements with a calibrated integrating sphere:

$$R\left({\rm{W}}\,{{\rm{m}}}^{-2}\,{{\rm{sr}}}^{-1}\,{{\rm{nm}}}^{-1}\right)=F\left({\rm{DN}}\right)$$

The results of the analyses of acquired calibration data show that using a B-spline as a model for the calibration curve it is possible to obtain the best fit of experimental data.

The characterization at pixel level was performed, giving for LUKE 3 × 2,048 × 1,088 calibration curves (one curve per pixel for each RGB Bayer filter).

The calibration of the acquired scientific images starts from the raw data (acquired frames), the detector temperature (in housekeeping data) and the integration time of the image together being used for calculating the bias frame. This bias frame, composed of the sum of the dark signal and the fixed pattern noise, is subtracted from the raw image.

The three colour frames given by the Bayer filter are then retrieved after applying the debayering algorithm.

The pixel value in DN of the obtained frames is then converted to radiance (W m^−2^ sr^−1^ nm^−1^) by applying the calibration curves obtained by on-ground calibration and confirmed by in-flight check before the fly-by of the Didymos system. Final calibrated images include three separate planes associated with the three RGB filters produced by the debayering process.

### Dimorphos shape constraints

The overall size of Dimorphos, as viewed by LICIACube, can be retrieved by combining images in which the lit side of the moonlet is visible in a following subset of images, obtained just after the CA and showing the outline of the dark side of Dimorphos (Extended Data Fig. 4).

Two pairs of images, in which both the illuminated and non-illuminated hemispheres can be seen independently, are used to perform this analysis. Each pair of images is acquired inside the same acquisition triplet and therefore they have very similar observation geometries.

In the short-exposure images (exposure time 0.7 ms), the illuminated hemisphere is clearly visible, whereas in the long-exposure ones (exposure time 35 ms) the non-illuminated part of the asteroid appears as a shadow in the saturated part of the plume.

By knowing the distance between the spacecraft and the target (with an accuracy of about 2 km at CA), the pixel scale in metres is determined for all the exploited images. After choosing a signal threshold so that the plume and Dimorphos are seen as different objects, a classical computer vision algorithm enables the determination of the object sizes. Considering the Dimorphos axes values computed using the DART measurements (that is, *x* = 177 m, *y* = 174 m and *z* = 116 m) (ref. ^1^) and taking into account that roughly a half of the hemisphere area can be visible in each of the selected images, one object per each image with size between 3,000 m^2^ and 6,000 m^2^ is selected. Furthermore, in one image it is also possible to extract the orientation of the objects and, hence, the axis sizes.

In particular, by looking at Extended Data Fig. 4, the values of the semi-axis A1 = 80 m and of the axis A2 = 100 m are determined with an uncertainty of 14 m, in good agreement with what was found by DART, taking into account that the entire shape is not determined by this single analysis.

### Cone geometry methods

Equation (1) gives the geometric relation between a perfectly axisymmetric cone and its projection onto a plane in Euclidean space, where *α* is the half aperture angle of the original cone, *δ* is the half angle of the projected cone and *θ* is the angle between the axis of the original cone and the plane onto which it is projected (Extended Data Fig. 2).

1 $$\tan \delta =\frac{\tan \alpha }{\sqrt{{\cos }^{2}\theta -{\tan }^{2}\alpha {\sin }^{2}\theta }}$$

The projected aperture angles (2*δ*) are measured using LUKE images, and the SPICE data enable the calculation of camera planes in the inertial space. These are the planes to which the images are projected at each image acquisition time. Extended Data Table 1 details the image parameters used, and Extended Data Fig. 1 shows cropped portions of the respective images, which were used for the measurement of the projected aperture angle 2*δ*. The uncertainty of the measurements is the minimum measurement possible by the protractor used, which is 1°.

#### Deriving an upper limit for the aperture angle

Equation (1) is rewritten as equation (2) for distinction. Equation (2) implies that given a measured projected half angle *δ* of a cone, the highest possible half angle α of the original cone can be obtained when the angle between the cone axis and the projected plane is 0°. A static cone is assumed over all six observations. The lowest projected aperture angle measured is the highest possible value of the original cone aperture angle. As such, the upper limit for the aperture angle of the ejecta cone has to be 140° with an uncertainty of 1°.

2 $$\tan \alpha =\frac{\tan \delta \cos \theta }{\sqrt{1+{\tan }^{2}\delta {\sin }^{2}\theta }}$$

#### Constraining the axis and the aperture angle of the ejecta cone

Using these measured data and SPICE data, a nonlinear equation for each observation of the cone is constructed. A projected plane is defined by introducing the following equation, *ax* + *by* + *cz* + *d* = 0, where *a*, *b*, *c* and *d* are the coefficients describing the plane and *x*, *y* and *z* are the coordinates. The unit vector of the cone axis is also defined as (*p*, *q* and *r*). As using these geometric constraints yields *θ*, *θ* in equation (1) can be replaced with the quantities defined above and rewritten in the following way:

3 $$f=-{\tan }^{2}\alpha +{\tan }^{2}\delta \left(1-\frac{{(a\times p+b\times q+c\times r)}^{2}}{{k}^{2}}(1+{\tan }^{2}\alpha )\right)=0$$

where *k* is (*a*^2^ + *b*^2^ + *c*^2^)^1/2^. This equation is the constraint that the cone geometry must satisfy.

In equation (3), there are four knowns from measurements (*δ*, *a*, *b* and c), whereas others (*α*, *p*, *q* and *r*) are unknown. Note that *α* can be constrained based on the above discussion. Thus, it is necessary to have four equations to solve *p*, *q*, *r* and tan^2^*α*, where *α* is eventually calculated. Five equations derived from the above format and the equation of the unit vector components lead to six equations in total. As four terms must be solved, all the 15 combinations are tried choosing four from six equations. The following equations are a possible combination that includes the unit vector equation.

$${f}_{1}=-{\tan }^{2}\alpha +{\tan }^{2}{\delta }_{1}\left(1-\frac{{(ab{c}_{10}\times p+ab{c}_{11}\times q+ab{c}_{12}\times r)}^{2}}{{k}_{1}^{2}}(1+{\tan }^{2}\alpha )\right)=0$$

$${f}_{2}=-{\tan }^{2}\alpha +{\tan }^{2}{\delta }_{2}\left(1-\frac{{(ab{c}_{20}\times p+ab{c}_{21}\times q+ab{c}_{22}\times r)}^{2}}{{k}_{2}^{2}}(1+{\tan }^{2}\alpha )\right)=0$$

$${f}_{0}=-{\tan }^{2}\alpha +{\tan }^{2}{\delta }_{0}\left(1-\frac{{(ab{c}_{00}\times p+ab{c}_{01}\times q+ab{c}_{02}\times r)}^{2}}{{k}_{0}^{2}}(1+{\tan }^{2}\alpha )\right)=0$$

$${f}_{4}={p}^{2}{+q}^{2}+{r}^{2}-1=0$$

$${k}_{0}^{2}={ab}{c}_{00}^{2}+{ab}{c}_{01}^{2}+a{c}_{02}^{2}$$

As an additional check, synthetic cones at known random axes with an aperture angle of 140° are generated and observed at different camera positions such that they could be viewed through a side-on profile, similar to the LUKE images. The plane geometry coefficients (*a*, *b*, *c*) that define the camera plane in inertial space are used to compute the projected aperture angles (2*δ*) for three camera positions. Then, the three nonlinear equations that were created by the synthetic cone generation and the unit vector equation are numerically solved, to find the four needed unknowns. The optimize.roots routine of the python library scipy^19^, which can be initiated with guesses of the cone axis and of the aperture angle 2*α*, is used for solving this system of nonlinear equations. Given the nonlinear nature of the equations, the guess of the angle is converted to tan^2^*α*, before initiating the solving routine. A series of starting point guesses are computed combining different directions for the axis solution and an angle for the aperture angle. The vectorial part of the guess is thus based on systematically sampling all the possible directions around a unit hemisphere with enough resolution using a spherical coordinate system. The guess for the angle of the solution is thus appended with all the sampled directions and iterated over all the guess combinations. As such, visualizing the results for the solved axis and the aperture angle using several plots, a solution for the original axis of the synthetic cone is recovered to an accuracy of angular separation of less than 0.1°. The solution for the aperture angle has an accuracy of less than 0.2°.

As there are several ways of choosing a combination of equations to be solved, a unique solution is not obtained for the cone axis. Therefore, the axis solution needs to be rotated in three-dimensional space such that the rotated cone axis matches with the position angle (angle measured from the projected north pole of the celestial sphere towards the east in the LUKE plane) of the observed ejecta cone axis in images. It is noteworthy in this context that a twist angle of 15° has to be applied to image planes before proceeding to a geometrical analysis of the position angle because of the imprecisions in the currently available LICIACube SPICE data. Following this twist-angle correction, first, the rotation required in the LUKE plane for the projection of the solved cone axis to match the position angle of the ejecta cone axis in images is found. Next, the solved cone axis is rotated along the LUKE boresight in three-dimensional space in very small angular (0.18°) increments up to 360°. At each increment, the new axis is projected onto the LUKE plane to find its angular separation with respect to the position angle of the ejecta cone axis in the images. Therefore, the resulting solution reaches the new axis with the least angular separation with respect to the position angle of the ejecta cone axis in images, when projected to the LUKE plane. The position angle of the ejecta cone was measured using the image reported with ID 1 in Extended Data Fig. 1.

Once a candidate solution axis is obtained, which matches the position angle of the ejecta cone in images, the ejecta cone is simulated at the timestamps of five images used for this analysis at their observation geometries, in which the images were initially acquired (Extended Data Fig. 1). Image ID (6) in Extended Data Fig. 1 is used to reject or accept candidate solutions, because of its very different observing geometry, compared with other images. Going through all the 15 combinations of the equations, all the candidate solutions, obtained after matching the positional angle of the ejecta cone in the image ID 1 in Extended Data Fig. 1, are explored. An approach similar to that in ref. ^20^ is applied to show the range of solutions for the cone axis direction that are mathematically possible and the derived solution constrained by different view geometries (Extended Data Fig. 3). The solution is a 144°-aperture angle cone with its axis pointing to (RA, DEC) = (137°, +19°). This solution is obtained by solving for the combination of three nonlinear equations formed by images ID (2), (4) and (5) in Extended Data Fig. 1 and the unit vector equation. The obtained aperture angle of 144° exceeded the upper limit of 140° placed above because image ID 1 in Extended Data Fig. 1 does not go into solving this specific combination of equations. Accordingly, the aperture angle of the ejecta cone is established as 140 ± 4°. The position angle of the axis solution in image ID 1 in Extended Data Fig. 1 is 72° once considered the twist angle of 15° needed to account for the imprecisions in SPICE data. The angular separation between the cone axis and the incoming DART direction is 10°.

Because of the 15° twist angle required to account for the SPICE imprecisions, the position angle of the ejecta cone in image ID 1 in Extended Data Fig. 1 oscillates between 105° and 75°. Consequently, the uncertainty of the cone axis oscillates between RA: 128°, 145° and DEC: +29°, +7°. Therefore, this results in an axis solution of (RA, DEC) $=\,{{137}_{-9}^{+8}}^\circ ,\,+{{19}_{-12}^{+10}}^\circ$.

### Filamentary streams

To understand the morphology of the ejecta and spatial reference, filamentary streams are labelled in the highest spatially resolved image acquired just before the CA (Fig. 2). Filamentary streams are defined as rectilinear extended structures extending from the surface of Dimorphos. They are connected to ray crater systems (see ref. ^21^ and references therein), and may constrain the boulder-rich surface morphology of the target, internal structure and shape for the impact and ejecta modelling in the future^8,22,23^.

Using DART, LICIACube and Dimorphos referencing positions calculated through reconstructed SPICE data, 18 filaments can be distinguished extending across the image up to 4 km at an exposure time of 10 ms (Fig. 2). The streams are arising nearly radially from the photocentre of the ejecta.

#### Upper limits on ejection velocities from early structures

Ejecta velocities are determined from a pair of sequential frames, indexed *k* − 1 and *k* and separated in time by ∆*t*, beginning with the angular projection measured at the field of view of the instrument. From each observation, spacecraft position S, ejecta origin position O, distance from spacecraft to ejecta origin position D, angular separation of ejecta structure from origin *θ* and projected ejecta structure extension *P*_*j*_ are defined (see Extended Data Fig. 5a for the labelling). These projected ejecta velocities can be used to estimate the magnitudes of the ejecta velocities when the observations fulfil certain conditions. Assuming that the angle *ω* is virtually unchanged between the sequential frames, it is possible to postulate

4 $$\frac{{\sigma }_{k}}{{\sigma }_{k-1}}=\frac{{Pj}_{k}}{{Pj}_{k-1}}=\frac{\Delta {t}_{k}}{\Delta {t}_{k-1}}\,$$

The projected ejecta structure extension is given as

5 $${Pj}_{k}=2({D}_{k}\pm {\sigma }_{k})\tan \left(\frac{{\theta }_{k}}{2}\right)$$

Thus, solving for *σ*_*k*_ as a function of the known quantities and *σ*_(*k*−1)_:

6 $${\sigma }_{k}=\left|\frac{\left(\frac{\Delta {t}_{k}}{\Delta {t}_{k-1}}\right){D}_{k-1}{{\rm{FOV}}}_{k-1}-{D}_{k}{{\rm{FOV}}}_{k}}{{{\rm{FOV}}}_{k-1}\pm {{\rm{FOV}}}_{k}}\right|$$

7 $${{\rm{FOV}}}_{k}=\tan \left(\frac{{\theta }_{k}}{2}\right)$$

Finally, substituting these quantities into the cosine law from the triangles defined in Extended Data Fig. 5a,

8 $${P}_{k}^{2}={V}^{2}{\Delta }^{2}{t}_{k}={D}_{k}^{2}+{({D}_{k}\pm {\sigma }_{k})}^{2}-2({D}_{k}\pm {\sigma }_{k}){D}_{k}\cos ({\theta }_{k})$$

where *V* is the true magnitude of the observed velocity. The projection angle is also solved:

9 $$\cos (\omega )=\frac{{\sigma }_{k}-{P}_{k}-{{Pj}}_{k}}{-2{P}_{k}{{Pj}}_{k}}$$

Solving equations (8) and (9) yields two solutions. The solution that yields coherent velocity through different sequential frames—that is, the same order of magnitude and smallest standard deviation, is kept and shown in Extended Data Table 2. Errors are propagated based on an average manual error of 3 pixels when measuring the projected distances.

The Didymos system orbital configuration, DART trajectory, LICIACube trajectory and relative positioning and instrument framing are calculated through reconstructed SPICE data.

#### Resolved morphological features and ejection velocities

The morphological features are tracked according to their visual distinctiveness between the frames taken 106 s (*D*_Dimo_ = 376 km) and 118 s (*D*_Dimo_ = 304 km) after the impact. The features are classified according to their apparent morphology: C, clumps; N, bright nodules; and B, filament breaking, merging, discontinuities and undulations (Fig. 3). Their orientation is tracked with respect to the filamentary streams, because many features are observed along their extension from the surface to the solar system environment, or in between.

Both solutions are provided for the estimation of the velocity magnitudes in Extended Data Table 2. As all features are studied in only two frames, it is impossible to distinguish between any preferential solution.

### RGB analysis methods

The RGB capabilities of the LUKE camera enable colour investigation of the plume ejected by Dimorphos. Whereas on rocky surfaces the differences in colours are related mostly to composition and alterations because of space weathering^24^, in diffuse ejecta plumes such as those observed by LICIACube, other effects can lead to colour changes because of physical properties of particles, such as the presence of extremely small grain sizes^25^.

Triplets of images with different exposure times were acquired during the fly-by. The last triplet in which Dimorphos and the plume generated by DART impact are still almost entirely visible is used for colour investigation. The triplet is composed of images acquired at 2022-09-26 23:17:03.000 (0.5 ms exposure time), 2022-09-26 23:17:03.004 (4 ms exposure time) and 2022-09-26 23:17:03.024 (20 ms exposure time). For reference on the wavelength range covered by the RGB filter, see ref. ^26^. On the calibrated images, the background is first evaluated to perform the removal of all areas that are not characterized by the presence of a plume. An average value of the background is calculated in the area diametrically opposite to the position of the binary system. Thus, the signal-to-noise ratio is computed for each channel in each image (Extended Data Fig. 6).

At the end of this process, the pixels in which the signal-to-noise ratio is less than 10 are masked. Before evaluating the channel ratios, the solar contribution is removed from the LUKE filters (*R* = 0.1320, *G* = 0.1706 and *B* = 0.1569). The maps resulting from the ratio of the three filters together with the associated uncertainties are shown in Extended Data Fig. 7.

## Online content

Any methods, additional references, Nature Portfolio reporting summaries, source data, extended data, supplementary information, acknowledgements, peer review information; details of author contributions and competing interests; and statements of data and code availability are available at 10.1038/s41586-023-06998-2.

## Figures and Tables

**Extended Data Table 1:**
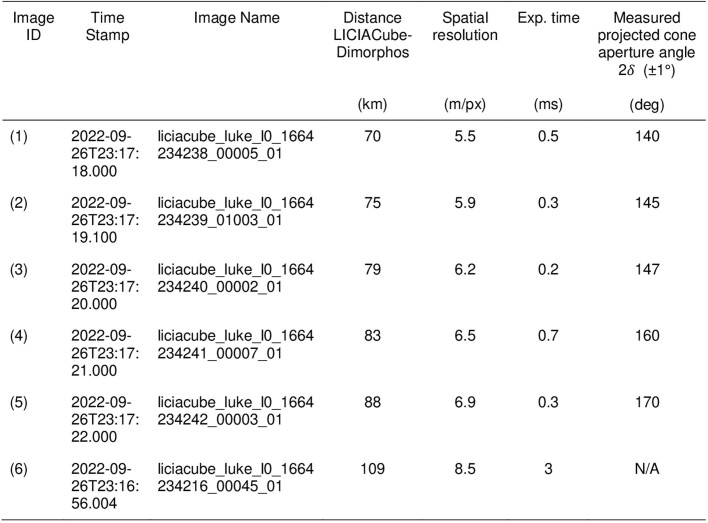
Sample of LUKE images used for the determination of the cone geometry

Note that the Image IDs are in order of distance from Dimorphos, not in chronological order.

**Extended Data Table 2:**
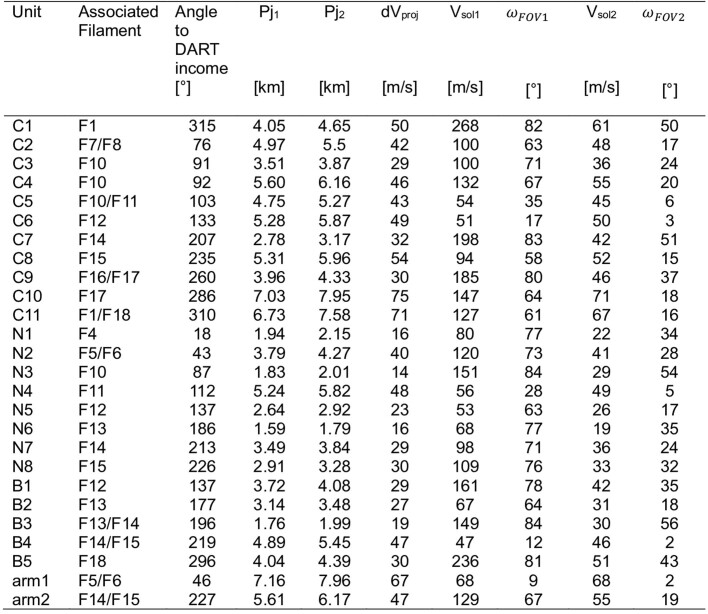
Morphological features and ejection velocity estimation

Projected length errors are roughly ±0.1 km. Projected Velocity errors are roughly ±5 m/s. Magnitude velocity errors are estimated to ±10 m/s.

**Extended Data Table 3:**
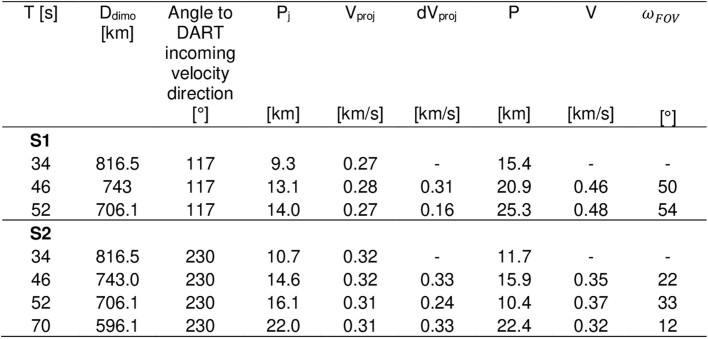
Velocities from the early ejected structures detected in the first frame observed by the LUKE instrument at 34 s after the impact

D_Dimo_ is the distance between LICIACube and Dimorphos by the SPICE data. Projected Length P_j_ errors are roughly ±0.15 km. Projected Velocity dV_proj_ errors are roughly ±0.015 km/s. Magnitude velocity V errors are estimated to be ±0.05 km/s.

## Data Availability

The data files for the Dimorphos viewing geometry are available at NAIF (https://naif.jpl.nasa.gov/naif/data.html). All the raw and calibrated LICIACube data, together with the needed calibration files and documentations, are publicly released through the Planetary Data System (PDS) (https://pds-smallbodies.astro.umd.edu/data_sb/missions/dart/index.shtml) as a separate bundle with respect to DART ones. These data are already publicly available at the ASI-SSDC LICIACube SOC (https://www.ssdc.asi.it/liciacube), and LUKE images acquired over Didymos can be also analysed using the SSDC webtool MATISSE (https://tools.ssdc.asi.it/Matisse/).

## References

[1] Daly RT (2023). Successful kinetic impact into an asteroid for planetary defence. Nature.

[2] Graykowski A (2023). Light curves and colours of the ejecta from Dimorphos after the DART impact. Nature.

[3] Li J-Y (2023). Ejecta from the DART-produced active asteroid Dimorphos. Nature.

[4] Thomas CA (2023). Orbital period change of Dimorphos due to the DART kinetic impact. Nature.

[5] Cheng AF (2023). Momentum transfer from the DART mission kinetic impact on asteroid Dimorphos. Nature.

[6] Dotto E (2021). LICIACube—The Light Italian Cubesat for Imaging of Asteroids In support of the NASA DART mission towards asteroid (65803) Didymos. Planet. Space Sci..

[7] Dotto E, Zinzi A (2023). Impact observations of asteroid Dimorphos via Light Italian CubeSat for imaging of asteroids (LICIACube). Nat. Commun..

[8] Rossi A (2022). Dynamical evolution of ejecta from the DART impact on Dimorphos. Planet. Sci. J..

[9] Bradski G (2000). The OpenCV library. Dr Dobbs J. Softw. Tools.

[10] Feldman PD (2007). Hubble Space Telescope observations of Comet 9P/Tempel 1 during the Deep Impact encounter. Icarus.

[11] Lara LM (2007). Behavior of Comet 9P/Tempel 1 around the Deep Impact event. Astron. Astrophys..

[12] Marchi S, Brunetto R, Magrin S, Lazzarin M, Gandolfi D (2005). Space weathering of near-Earth and main belt silicate-rich asteroids: observations and ion irradiation experiments. Astron. Astrophys..

[13] Bertini I (2009). Activity evolution, outbursts, and splitting events of comet 73P/Schwassmann-Wachmann 3. Astron. Astrophys..

[14] Levasseur-Regourd A-C (2018). Cometary dust. Space Sci. Rev..

[15] Wooden, D., Desch, S., Harker, D., Gail, H.-P. & Keller, L. in *Protostars and Planets V* (eds Reipurth, B. et al.) 815–833 (Univ. Arizona Press, 2007).

[16] Combi MR (1994). The fragmentation of dust in the innermost comae of comets: possible evidence from ground-based images. Astron. J..

[17] Clark BC (2004). Release and fragmentation of aggregates to produce heterogeneous, lumpy coma streams. J. Geophys. Res..

[18] Naidu SP (2020). Radar observations and a physical model of binary near-Earth asteroid 65803 Didymos, target of the DART mission. Icarus.

[19] Chen H-S, Stadtherr M (1981). A modification of Powell’s dogleg method for solving systems of nonlinear equations. Comput. Chem. Eng..

[20] Farnham TL, Cochran AL (2002). A McDonald Observatory study of Comet 19P/Borrelly: placing the Deep Space 1 observations into a broader context. Icarus.

[21] Sabuwala T (2018). Ray systems in granular cratering. Phys. Rev. L..

[22] Raducan S, Davison TM, Collins GS (2022). Ejecta distribution and momentum transfer from oblique impacts on asteroid surfaces. Icarus.

[23] Fahnestock G (2022). Pre-encounter predictions of DART impact ejecta behavior and observability. Planet. Sci. J..

[24] Clark, B. E., Hapke, B., Pieters, C. & Britt, D. in *Asteroids III* (eds Bottke, W. F. Jr et al.) 585–600 (Univ. Arizona Press, 2002).

[25] Meech KJ (2005). Deep Impact: observations from a worldwide Earth-based campaign. Science.

[26] Poggiali G (2022). Expected investigation of the (65803) Didymos–Dimorphos system using the RGB spectrophotometry data set from the LICIACube Unit Key Explorer (LUKE) wide-angle camera. Planet. Sci. J..

